# Endometrial factors similarly induced by IFNT2 and IFNTc1 through transcription factor FOXS1

**DOI:** 10.1371/journal.pone.0171858

**Published:** 2017-02-15

**Authors:** Kazuya Kusama, Rulan Bai, Keigo Nakamura, Sayaka Okada, Jiro Yasuda, Kazuhiko Imakawa

**Affiliations:** 1 Animal Resource Science Center, Graduate School of Agricultural and Life Sciences, The University of Tokyo, Ibaraki, Japan; 2 Department of Emerging Infectious Diseases, Institute of Tropical Medicine (NEKKEN), Nagasaki University, Nagasaki, Japan; University of Missouri Columbia, UNITED STATES

## Abstract

In ruminants, Interferon tau (IFNT) is the pregnancy recognition protein produced by the mononuclear trophectoderm of the conceptus, and is secreted into the uterine lumen during the peri-attachment period. In our previous study, the high-throughput RNA sequencing (RNA-seq) data obtained from bovine conceptuses during the peri-attachment period identified two *IFNT* mRNAs, *IFNT2* and *IFNTc1*. However, how each of these IFNT variants regulates endometrial gene expression has not been characterized. Using RNA-seq analysis, we evaluated how IFNT2 and IFNTc1 affected transcript expression in primary bovine endometrial epithelial cells (EECs). IFNT treatment induced 348 differentially expressed genes (DEGs); however, there are few DEGs in IFNT2 or IFNTc1 treated EECs, indicating that IFNT2-induced DEGs were similar to those induced by IFNTc1 treatment. In in silico analysis, we identified four IFNT2- and IFNTc1-induced pathways: 1) type II interferon signaling, 2) proteasome degradation, 3) type III interferon signaling, and 4) DNA damage response. We further demonstrated that IFNT2 and IFNTc1 up-regulated several transcription factors, among which forkhead box S1 (*FOXS1*) was identified as the most highly expressed gene. Furthermore, the knockdown of *FOXS1* in IFNT2- or IFNTc1-treated EECs similarly down-regulated 9 genes including *IRF3* and *IRF9*, and up-regulated 9 genes including *STAT1*, *STAT2*, and *IRF8*. These represent the first demonstration that effects of each IFNT on EECs were studied, and suggest that endometrial response as well as signaling mechanisms were similar between two IFNT variants existed in utero.

## Introduction

Interferon tau (IFNT), classified as a type I IFN along with IFN alpha, IFN beta and IFN omega, is the pregnancy recognition protein in all ruminants [1]. IFNT, produced by the mononuclear trophectoderm of the conceptus, is secreted into the uterine lumen during the peri-attachment period [1, 2], although a few studies indicate that IFNT is detected in uterine vein serum [3, 4]. IFNT down-regulates the expression of endometrial oxytocin receptors and then maintains the corpus luteum function via inhibition of the luteolytic pulse of endometrial prostaglandin F2α [5–7]. Bovine IFNT increases on day 15 of pregnancy (day 0 = day of estrus), peaks on days 19–20 (conceptus attachment to endometrium begins on days 19–19.5), and is decreased soon after the initiation of conceptus attachment to the uterine epithelium [8–11].

To determine the number of IFNT genes expressed in the bovine uterus, several studies with the use of PCR have previously been executed [12, 13]. We also utilized the high-throughput RNA sequencing (RNA-seq) analysis, identifying two *IFNT* transcripts, *IFNT2* and *IFNTc1*, in RNAs obtained from pregnant days 17, 20, and 22 bovine conceptuses [9, 14]. In addition, we showed that *IFNT2* expression *in utero* was five-fold higher than that of *IFNTc1* [9]. Over the past decade, various global analyses were performed to study the expression of transcripts in bovine endometrium [15–20]. These observations found changes in dynamic gene expression in pregnant and cyclic endometria, and identified optimal transcript and/or protein expression *in utero* for maintaining pregnancy at several stages in early pregnancy. In addition, those studies demonstrated the effects of IFNT and/or progesterone on differentially expressed genes in bovine endometrium. In the previous studies [12, 13], IFNT variants identified were subjected to assays of antiviral or anti-luteolytic activity; however, how each of those IFNT variants affects endometrial gene expression has not been characterized.

Our previous studies showed that the upstream region of *IFNTc1* gene did not possess the JUN-binding site found in the *IFNT2* gene, and TEAD2 increased transcriptional activity of *IFNT2* only, resulting in the differential expression between *IFNT2* and *IFNTc1* in in vitro and possibly in vivo [21, 22]. In addition, both IFNT2 and IFNTc1 up-regulated IFN-stimulated genes (ISGs), including ISG12, ISG15, or MX dynamin-like GTPase (MX)1, while only IFNTc1 up-regulated the expression of MX2 in bovine endometrial epithelial cells [23]. We therefore hypothesized that effects of IFNT2 and IFNTc1 differ in the bovine endometrium. In this study, we evaluated how IFNT2 and IFNTc1 affected primary bovine endometrial epithelial cells using RNA-seq, followed by quantitative PCR analysis.

## Materials and methods

### Cell preparation, culture condition

In this study, we did not perform any animal experiments. Bovine uterine endometrial epithelial cells (EECs) were collected from Holstein cows at local abattoir (Tsuyama Meat Center) in accordance with protocols approved by local institutional animal care [24], and the protocol for bovine cell cultures was approved by the Ethics Committee of the University of Tokyo (Permit Number: 449–2126). In brief, uteri of the early luteal phase (days 2 to 5) were used in this study. The hysterectomized uterine lumen was trypsinized (0.3% w/v) in order to detach the epithelial cells and then EECs were isolated. The isolated EECs were cultured on collagen type I-coated culture dish in DMEM/F12 (1:1) medium supplemented with 10% (v/v) FBS, 40 units/ml of penicillin, and 40 μg/ml of streptomycin at 37°C under 5% CO_2_ in humidified air [11]. Human 293T cells (CRL-3216, ATCC) were grown in DMEM supplemented with 10% (v/v) FBS and antibiotics at 37°C in 5% CO2 [23].

### Production and purification of recombinant IFNs

293T cells were transfected with the expression plasmid for IFNT2, IFNTc1 or IFNA [23] and culture media were collected at 48–72 hours after transfection [23]. Recombinant IFNs secreted to culture media from cells were purified using His-tagged protein purification reagent (Medical and Biological Laboratories, Nagoya, Japan) according to the manufacturer’s instructions [23]. The titers of purified recombinant IFNT2, IFNTc1, and IFNA were determined by the assay using MDBK cells and VSV as previously reported [25].

### RNA extraction and preparation for RNA-seq analysis

RNA was extracted from cultured EECs using Isogen (Nippon gene, Tokyo, Japan) according to the manufacturer’s instructions. A portion of total RNA from cultured EECs treated with or without IFNT2 or IFNTc1 (n = 3 each) was pooled. High-throughput sequencing libraries were prepared using the SureSelect Strand Specific RNA Library Prep Kit (Agilent Technologies, Santa Clara, CA) according to the manufacturer’s instructions, and analysis was performed by Kazusa DNA Research Institute (Chiba, Japan). Primary sequencing data were deposited to the DDBJ (DNA Data Bank of Japan) Sequence Read Archive (accession number DRA005460).

### Mapping reads to the bovine genome

Nucleotide sequences identified by RNA-seq analysis were trimmed by PRINSEQ-lite v0.19.2. Trimmed sequences were generated as FASTQ outputs and analyzed on the basis of the TopHat/Cufflinks pipeline based on the bovine genome (bosTau8) and reference annotations obtained from UCSC genome browser (http://genome.ucsc.edu). Differential and significant gene expression analysis was performed with the use of gene-level FPKM (fragments per kilobase of gene locus summarized mRNA per million reads) expression levels. Genes were selected with the criteria of an absolute expression level >10 FPKM in either IFNT2- or IFNTc1-treated samples with at least 1.5-fold higher expression in IFNT2 or IFNTc1 than non-treated EECs.

### RNA extraction and quantitative RT-PCR

Using ISOGEN reagent (Nippon gene), total RNAs were extracted from cultured EECs treated with IFNTs, which were performed three times independently. For real-time PCR analyses, isolated RNA (total 0.5 μg) was reverse transcribed to cDNA using the ReverTra Ace qPCR RT Kit (Toyobo, Osaka, Japan) according to the manufacturer’s instructions. The cDNA reaction mixture was subjected to real-time PCR amplification using the Thunderbird SYBR qPCR Mix Kit (Toyobo) with primers listed in S1 Table, and PCR amplification was carried out on a Step One Plus real-time PCR System (Applied Biosystems, Foster City, CA). Amplification efficiencies of each target and the reference gene, bovine glyceraldehyde-3-phosphate dehydrogenase (*GAPDH*), were examined through their calibration curves and found to be comparable. The thermal profile for qPCR consisted of 40 cycles at 95°C for 15 sec, and annealing and extension at 60°C for 60 sec. Average threshold (Ct) values for each target were determined by Sequence Detection System software v2.2 (Applied Biosystems). Each run was completed with a melting curve analysis to confirm the specificity of amplification and the absence of primer dimer [11].

### Transfection of small interfering RNA

The nucleotide structures of *FOXS1* small interfering RNAs (siRNAs) were designed through the use of the siDirect program (RNAi, Tokyo, Japan), and all siRNAs were prepared commercially (Sigma–Aldrich). The nucleotide sequences of bovine FOXS1 (NM_001099716.1) were used to design the siRNA. EECs grown in 12-well plates were transfected with a nontargeting control siRNA (Invitrogen), FOXS1 #1 (5’-ACUCAAAGAAGAACAUUCCUG-3’, 5’-GGAAUGUUCUUCUUUGAGUGA-3’), or FOXS1 #2 (5’-AUGAUGUAGCGGUAGAUGCCG-3’, 5’-GCAUCUACCGCUACAUCAUGG-3’) siRNA (100 nM each) using Lipofectamine RNAiMAX reagent (3 μL, Invitrogen) according to the manufacturer’s instructions [22]. After the transfection, medium was removed, and the cells were cultured in fresh medium for 24 h. The EECs were treated with IFNT2 or IFNTc1. The concentrations for each siRNA were predetermined.

### Statistical analysis

Data are expressed as the mean ± SEM. Significance was assessed using the Dunnet comparisons test. A *P*-value < 0.05 was considered statistically significant.

## Results

### Differential gene expression between IFNT2- and IFNTc1-stimulated endometrial epithelial cells

**Fig 1:**
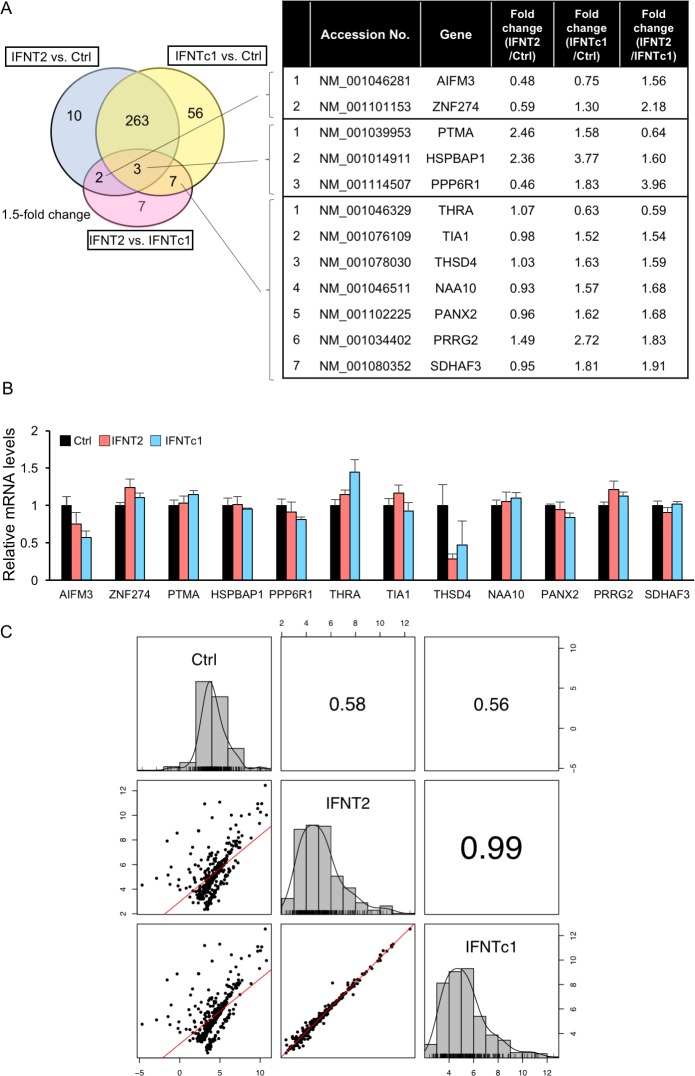
Differential gene expression in bovine endometrial epithelial cells treated with IFNT2 or IFNTc1. (A) Venn diagram shows the number of gene with 1.5-fold changes among Control (Ctrl), IFNT2, and IFNTc1 treatment groups, and right table lists increased or decreased genes in IFNT2 vs. IFNTc1 group, which overlap with Ctrl vs. IFNT2 or Ctrl vs. IFNTc1 group. (B) EECs were incubated without (Ctrl) or with IFNT2 or IFNTc1 (2 x 10^5^ cells/5000 IU/well) for 24 h. RNA was extracted from the EECs and subjected to real-time PCR analysis on mRNA expression with overlapping IFNT2 vs. IFNTc1 group with other groups. *GAPDH* mRNA was used as an internal control for RNA integrity. Value represent the mean ± SEM from three independent experiments in each treatment. (C) these diagrams show pair plots comparison among Ctrl, IFNT2, and IFNTc1, and density plots in each groups. Figures show correlation coefficient among Ctrl, IFNT2, and IFNTc1.

To investigate effects of IFNT2 and IFNTc1 on gene expression in EECs, RNA-seq analyses were executed, detecting 348 differentially expressed genes (DEGs) among non- (Ctrl), IFNT2- and IFNTc1-treatment groups (S2 Table). The venn diagram shows the number of genes with 1.5-fold changes among these groups, and the right table lists increased or decreased genes in IFNT2 vs. IFNTc1 group, which overlap with Ctrl vs. IFNT2 or Ctrl vs. IFNTc1 group (Fig 1A). Although RNA-seq analysis found that 12 gene expressions were increased or decreased, qPCR did not detect changes in those gene expressions (Fig 1B). A pair plot comparison of IFNT2 and IFNTc1 treatments showed that there were very few DEGs, which were confirmed with high degree of correlation coefficient, 0.99 (Fig 1C). These results indicated that IFNT2-induced DEGs were similar to those of IFNTc1 treatment in EECs.

**Fig 2:**
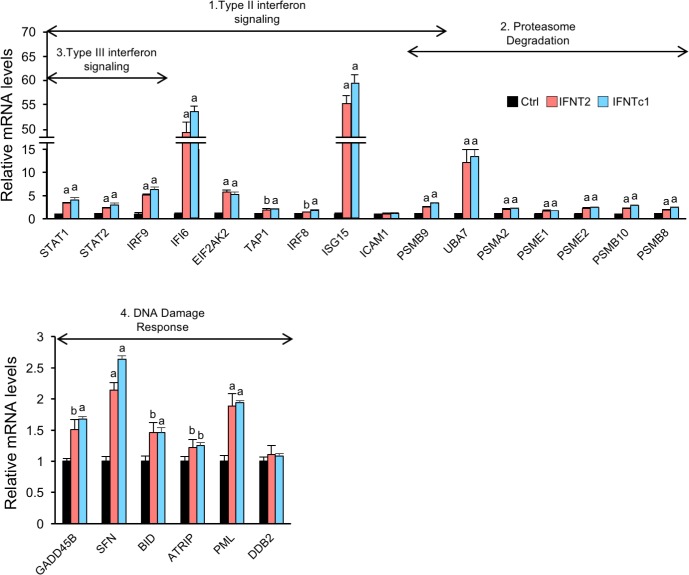
Identification of gene expression induced by IFNTs in EECs. EECs were incubated without (Ctrl) or with IFNT2 or IFNTc1 (2 x 10^5^ cells/5000 IU/well) for 24 h. RNA was extracted from the EECs and subjected to real-time PCR analysis to determine gene expression related to type II interferon, proteasome degradation, type III interferon, and DNA damage response signaling in Ctrl, IFNT2-, or IFNTc1-treated EECs (n = 3 each group). *GAPDH* mRNA was used as an internal control for RNA integrity. ^a^P < 0.01, ^b^P<0.05 vs. Ctrl. Value represent the mean ± SEM from three independent experiments in each treatment.

Increased DEGs from RNA-seq were then analyzed using the GO term (Enrichr; http://amp.pharm.mssm.edu/Enrichr/) and pathway analyses (WikiPathways; http://www.wikipathways.org/index.php/WikiPathways). These analyses detected 106 GO groups (S3 Table) and 4 pathways: 1) type II interferon signaling, 2) proteasome degradation, 3) type III interferon signaling, and 4) DNA damage response (Table 1). To further examine whether IFNTs regulated those 4 enriched pathways, all transcripts associated with these pathways were subjected to qPCR analysis. Transcripts except for *ICAM1* and *DDB2* were up-regulated by IFNT2 and IFNTc1, in agreement with those detected by the RNA-seq analysis (Fig 2).

**Table 1:** Genes related to IFNTs-induced enriched pathways in EECs.

	Pathway	P-value	Gene name
1	Type II interferon signaling (IFNG)	6.48E-07	STAT1, STAT2 IFI6 EIF2AK2 TAP1, IRF8, ISG15, IRF9, PSMB9, ICAM1
2	Proteasome Degradation	6.44E-03	UBA7, PSMA2 PSME1, PSME2, PSMB10, PSMB8, PSMB9
3	Type III interferon signaling	3.03E-02	STAT1, STAT2, IRF9
4	DNA Damage Response	3.25E-02	GADD45B, SFN, BID, ATRIP, PML, DDB2

### Determination of IFNTs downstream transcription factors

**Fig 3:**
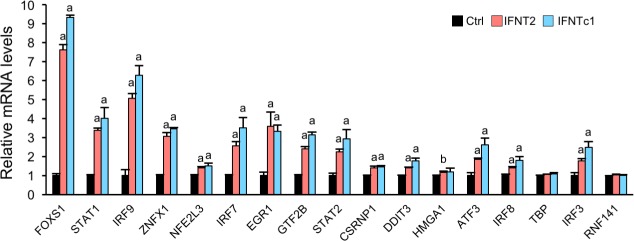
Determination of IFNTs’ downstream transcription factors. EECs were incubated without (Ctrl) or with IFNT2 or IFNTc1 (2 x 10^5^ cells/5000 IU/well) for 24 h. RNA was extracted from the EECs and subjected to real-time PCR analysis to determine the expression of transcription factors in Ctrl, IFNT2-, or IFNTc1-treated EECs (n = 3 each group). *GAPDH* mRNA was used as an internal control for RNA integrity. ^a^P < 0.01, ^b^P<0.05 vs. Ctrl. Value represent the mean ± SEM from three independent experiments in each treatment.

It is reported that IFNT binds its receptor, upon which transcription factors STAT1/2 and IRFs regulate the expression of interferon stimulated genes (ISGs) [15]. However, molecular mechanisms associated with IFNT-induced signaling pathway has not been well characterized. To identify transcription factors induced by IFNT stimulation in EECs, increased DEGs were subjected to GO term analysis. From the RNA-seq data, 17 transcription factors were identified as up-regulated DEGs (Table 2), among which 15 genes, *FOXS1*, *STAT1*, *IRF9*, *ZNFX1*, *NFE2L3*, *IRF7*, *EGR1*, *GTF2B*, *STAT2*, *CSRNP1*, *DDIT3*, *HMGA1*, *ATF3*, *IRF8*, *and IRF3* were up-regulated in IFNT2- or IFNTc1-treated EECs (Fig 3). In both RNA-seq and qPCR analyses, *FOXS1* exhibited the highest expression among these transcription factors.

**Table 2:** Transcriptional factors up-regulated by IFNTs in EECs.

	Accession No.	Gene	Fold change (IFNT2 /Ctrl)	Fold change (IFNTc1 /Ctrl)
1	NM_001099716	FOXS1	97.40	113.81
2	NM_001077900	STAT1	4.81	5.69
3	NM_001024506	IRF9	4.35	4.89
4	NM_001205716	ZNFX1	3.99	4.42
5	NM_001077899	NFE2L3	2.96	3.20
6	NM_001105040	IRF7	2.87	3.03
7	NM_001045875	EGR1	2.63	2.70
8	NM_001046142	GTF2B	2.48	2.82
9	NM_001205689	STAT2	2.47	1.93
10	NM_001098035	CSRNP1	2.06	2.19
11	NM_001078163	DDIT3	1.94	2.11
12	NM_001076523	HMGA1	1.80	2.03
13	NM_001046193	ATF3	1.66	1.80
14	NM_001083769	IRF8	1.64	1.62
15	NM_001075742	TBP	1.50	1.70
16	NM_001029845	IRF3	1.40	1.53
17	NM_001040566	RNF141	1.16	1.65

### Effects of FOXS1 knockdown on the expression of IFNTs downstream factors

**Fig 4:**
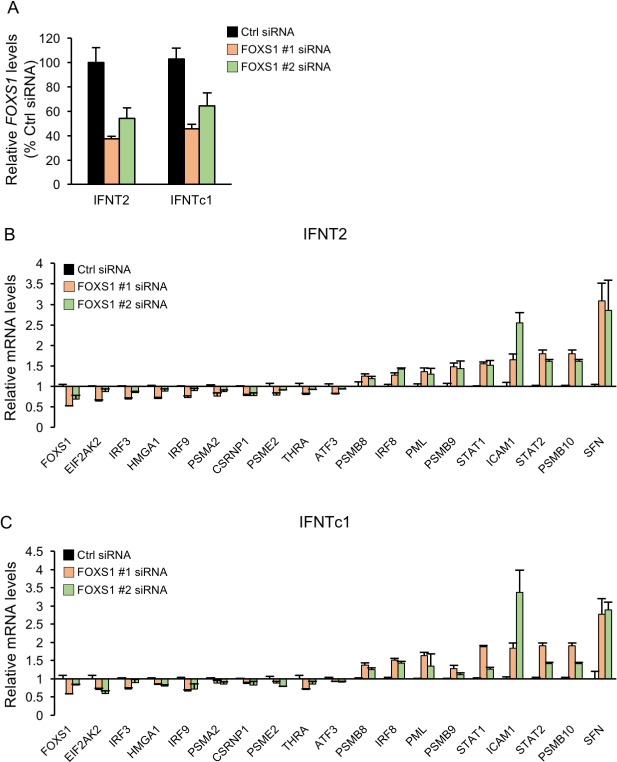
Effects of FOXS1 knockdown on the expression of IFNTs downstream factors. (A-C) EECs were transfected with non-targeting control (Ctrl: 200 nM) or *FOXS1* siRNA (#1 or #2: 200 nM) for 48h, and then incubated with IFNT2 (B) or IFNTc1 (C) (2 x 10^5^ cells/5000 IU/well) for 24 h. RNA was extracted from the EECs and subjected to real-time PCR analysis (n = 3 each group). *GAPDH* mRNA was used as an internal control for RNA integrity. Values represent the mean ± SEM from three independent experiments in each treatment.

To ascertain whether FOXS1 regulated the gene expression induced by IFNT2 or IFNTc1, we performed knockdown of FOXS1 by two siRNAs (#1 and #2), which specifically recognized *FOXS1* mRNA sequences in different regions (Fig 4A). Two *FOXS1* siRNAs down-regulated 9 genes, *EIF2AK2*, *IRF3*, *HMGA1*, *IRF9*, *PSMA2*, *CSRNP1*, *PSME2*, *THRA*, and *ATF3*, and up-regulated 9 genes, *PSMB8*, *IRF8*, *PML*, *PSMB9*, *STAT1*, *ICAM1*, *STAT2*, *PSMB10*, and *SFN* in IFNT2-treated EECs (Fig 4B). Similar to IFNT2 treatment, Two *FOXS1* siRNAs regulated 18 gene expressions in IFNTc1-treated EECs (Fig 4C).

## Discussion

In this study, we first demonstrated the global gene expression of primary bovine EECs treated with IFNT2 or IFNTc1, and identified a novel FOXS1 signaling pathway, resulting in IFNT response. Although several studies have shown variants of *IFNT* transcripts [9, 12, 13], their regulation and effects on EECs have not been characterized. In this study, IFNT2 and IFNTc1 induced transcripts associated with 4 enriched pathways. In addition, IFNT2 and IFNTc1 up-regulated several transcription factors, among which *FOXS1* was found as the highest expressed gene. Furthermore, the knockdown of *FOXS1* down-regulated 9 genes including *IRF3* and *IRF9*, and up-regulated 9 genes including *STAT1*, *STAT2*, and *IRF8*. These findings suggested that upon IFNT stimulation, FOXS1 could have mediated up- or down-regulation of IFNT-stimulated transcription factors such as STAT1, STAT2 and IRFs, followed by activation of type II interferon, proteasome degradation, type III interferon, and DNA damage response signaling pathways.

**Fig 5:**
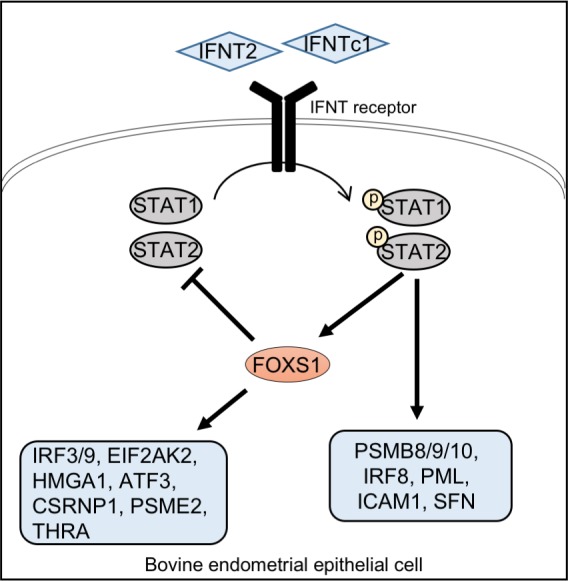
Diagram illustrating the potential role of IFNT through FOXS1 in EECs. IFNT2 and IFNTc1 bind to their receptor and then activate STAT1 or STAT2. Activated STATs up-regulate FOXS1 expression, which down-regulates STATs expression, resulting in a negative feedback loop between STATs and FOXS1.

FOXS1 is Forkhead type transcription factor expressed in Sertoli cells and peri-endothelial cells of the developing mouse fetal testis [26]. Male and female Foxs1 knockout mice are fertile, but the mutant males accumulate blood in the fetal testis [26]. Foxs1 is also expressed in gonadal-like cells in Gata6 conditional knockout mice [27]. In addition to gonadal-like cells, Foxs1 is expressed in neural crest derivatives [28–30]. However, molecular mechanisms on how FOXS1 regulates those phenotypic changes have not been characterized. In this study, *FOXS1* was up-regulated by IFNT2 and IFNTc1, and its knockdown regulated general IFNT-induced transcription factors such as STAT1 and STAT2 in bovine EECs. In addition, IFNA treatment similarly increased *FOXS1*, *STAT1* and *STAT2* expression (S1 Fig); however, type I IFNs such as IFNA and IFNB do not exist in the bovine and ovine uterine lumen during peri-implantation period. [12, 31]. In human dermal fibroblast cells, FOXS1, up-regulated by STAT4, induces the differentiation into myofibroblast [32]. In addition, we confirmed the presence of STAT1- or STAT2-binding elements on *FOXS1* promoter region (S2 Fig). These findings suggested that activation of STAT1 or STAT2 could induce FOXS1 expression and FOXS1 then down-regulate STAT1 and STAT2 expression: possible negative feedback loop between FOXS1 and STAT1/2 in the bovine endometrial epithelium (Fig 5).

In the pro-inflammatory response to control the immune system, proteasomes are replaced with immunoproteasomes, which are induced by interferon-gamma (IFNG) and increase the production of peptides for presentation of MHC class I molecules [33]. The proteasome consists of three units, 20S proteasome, 19S regulator, and proteasome activator 28 (PA28), among which PA28 is composed of PSME1 and PSME2 [33]. 20S proteasome has 20 subunits, among which 3 subunits are replaced with PSMB8, PSMB9, and PSME10 by IFNG stimulation, resulting in immunoproteasome formation. In our results, IFNT2 and IFNTc1 up-regulated not only *PSME1*, *PSME2*, *PSMB8*, *PSMB9*, and *PSMB10*, but also class I antigen presentation-related genes such as *BOLA-A*, *B2M*, *TAP1/2*, and several ubiquitin ligases (S2 Table). These results were consistent with our and other previous studies which demonstrated changes in protein and gene expression during peri-implantation periods [11, 20, 34]. In addition, FOXS1 knockdown increased the expression of *PSMB8*, *PSMB9*, and *PSMB10*. These findings suggested that IFNT2 and IFNTc1 induced immunoproteasome formation and class I antigen presentation in endometrial epithelial cells, which could condition the EECs for interaction with semi-allogenic conceptuses.

Our previous study demonstrated that *MX1*, *ISG12*, *ISG15*, *ISG17*, *IRF1*, and *IRF2* were similarly up-regulated by IFNT2 or IFNTc1 treatment, but *MX2* was only up-regulated by IFNTc1 [23]. This study showed that IFNTc1 tended to increase the expression of *MX2* compared with that of IFNT2. However, correlation coefficient of gene expression between IFNT2 and IFNTc1 was 0.99, indicating no or minimal difference in gene expression between IFNT2 and IFNTc1. The amino acid sequences of IFNT2 and IFNTc1 differ slightly, of which IFNTc1 has one casein kinase 2 phosphorylation domain whereas IFNT2 does not (S3 Fig). These results suggest that IFNT2 and IFNTc1 still differ in nucleotide structures and possibly functions, but further experiments are required to prove definitive functional differences between IFNT2 and IFNTc1.

In conclusion, this study demonstrated the global gene expression of IFNT2- or IFNTc1-treated primary bovine endometrial epithelial cell. One of these genes was transcription factor FOXS1, up-regulated by IFNT2 and IFNTc1, and its knockdown up-regulated STAT1 and STAT2. Therefore, FOXS1 could play a role as a negative feedback regulator of IFNTs signaling in bovine endometrial epithelial cells.

## Supporting information

S1 Fig Effect of IFNA on the expression of FOXS1 in bovine EECs. EECs were incubated without (Ctrl) or with IFNA (2 x 10^5^ cells/5000 IU/well) for 24 h. RNA was extracted from the EECs and subjected to real-time PCR analysis. *GAPDH* mRNA was used as an internal control for RNA integrity. ^a^P < 0.01 vs. Ctrl. Values represent the mean ± SEM from three independent experiments in each treatment.
(TIFF)

S2 Fig Possible STAT1- and STAT2-binding sites on *FOXS1* promoter region. (DOCX)

S3 Fig The amino acid sequences of IFNT2 and IFNTc1. (DOCX)

S1 Table Oligonucleotide primers for real-time PCR analyses. (XLSX)

S2 Table Lists of DEGs from RNA-seq analysis. (XLSX)

S3 Table Lists of GO term from RNA-seq analysis. (XLSX)
