# Supplementary figures and images for: Endometrial factors similarly induced by IFNT2 and IFNTc1 through transcription factor FOXS1
Source: PLoS One. 2017 Feb 15;12(2):e0171858. doi: 10.1371/journal.pone.0171858 (PMC5310909; doi:10.1371/journal.pone.0171858)

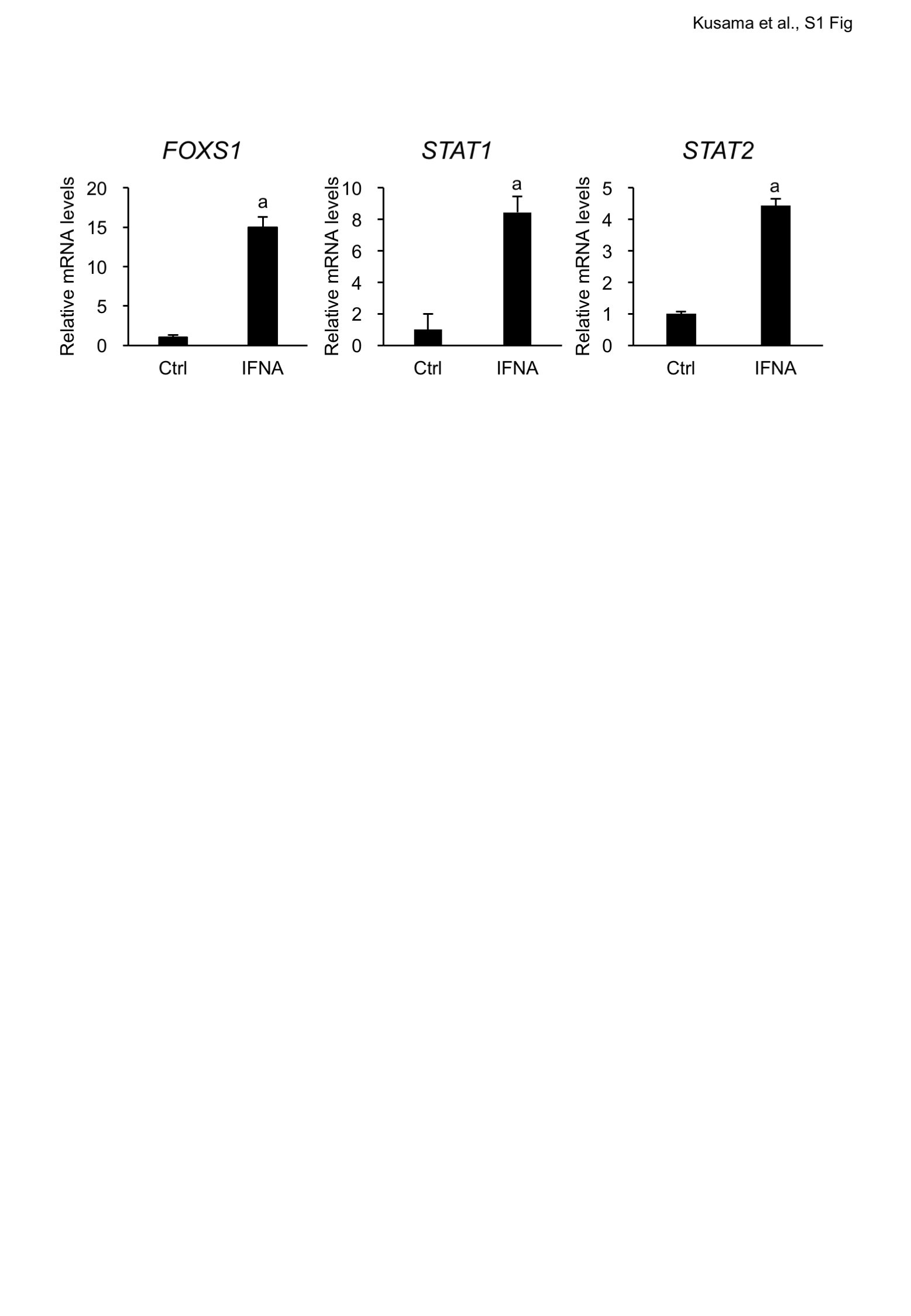

Supplement: S1 Fig — EECs were incubated without (Ctrl) or with IFNA (2 x 10^5^ cells/5000 IU/well) for 24 h. RNA was extracted from the EECs and subjected to real-time PCR analysis. GAPDH mRNA was used as an internal control for RNA integrity. aP < 0.01 vs. Ctrl. Values represent the mean ± SEM from three independent experiments in each treatment. (TIFF) [file pone.0171858.s001.tiff]
