# Supplementary material for: Endometrial factors similarly induced by IFNT2 and IFNTc1 through transcription factor FOXS1
Source: PLoS One. 2017 Feb 15;12(2):e0171858. doi: 10.1371/journal.pone.0171858 (PMC5310909; doi:10.1371/journal.pone.0171858)
Supplement: S2 Fig — (DOCX) [file pone.0171858.s002.docx]

**S2 Fig. Possible STAT1- and STAT2-binding sites on *FOXS1* promoter region.**

CTTTGCCACCGCCTGGACAACTGCAGAGTCATCAGCTCCATTTTAGAGATGGGAAACTGAGGCTCTGCCAGAGAATTGGTTGAGAGCCTCTTGGAAGGGGGATGTCTCTGGCCTGAGGTCTGTCCTCCCCATTCTAGTCACTAGAGGGCGCCAGCGCCCAGCAAGCCGGGTTTGGGCTGGCTCTCATGACCACCCCAGGTGCCCACGTCTTGCAGACCCCAGTGCTAGAAGGGCAGGGAACAGTGTGACCCCGAGTGCTCTGCACTTCGGGGAGGAGAAAGAGAATCACGGGCCTGGGAAGAGGAGGAAAGGAGAAAGAGGAAACCAGAGGTGGGGATTCAAGAGGCCCTGAGGCTCTTCTCACAAAGCTCCCTGTGCTTGAGCTCACAGATCCTACCTCTCCTCTCTCCTGAGAGTGTGTGTGTGTGTGTGTGTGTGTGTGTCTCCCTCGTCCTATCTTTGTGGATTTCTTTCTTTCTCTTGTTCAGTGATTCTTTTTCTGTGCTCTGTGCACGTGTGCACGTGCGCACGTGTATGGGGCTGGCTCTCAGTGTCTCCCCTTTTGCTGTCTACTTGCTGTCTCTTGTCTCTGCCTCTGTCTCCTGTGTGTTCCTGTCTCCCTATTATGTCCCCACCTCTCTTCGTCTCTGTCCTTCCTTTCTTCATTCTCTCTATCTCTGCCTTGATCTCTTTTTATTTGTCTCTGTCTCGCCCAGTTGCACATCCCATCCGTCCTCAACCACCACCACCAGGCAGCACATGTGGGCTGAGGGTCTCAGTCCCTTCCCTCCCATGTCTCCTCACCCTGGGCAGCTGCTCACAGCTTCCCAATCCTGGCCTCTCCTGCCCGCCCCTCTTGGGCCTGGGGGGAGGGGAGCGCGGGAGGAAGGGAGGGCAGCCTGGCGGGCCCACCCCTTTTTGCCTTTCCAGGCTGGGAGGCTGGGCCAGTGGGCCTTTTAACCAGCCTGGGCAGGCTGTGCCGGACACCAGCCCTGGACACCGTGCCTGGCCTCTGCGGTTCCGGCAGCCCGGCCAGCCCGGCCAGCCCGGCCAGC**ATG**CAGCAGCCACCCCCACCCGGGCCCCTGGCCCCTGCGGCCGAGCCAACCAAGCCTCCTTACAGCTACATCGCCCTGATCGCCATGGCCATCCAGAACTCTCCAGGGCAGCGGGCCACACTCAGCGGCATCTACCGCTACATCATGGGCCGCTTCGCCTTCTACCGCCACAACCGGCCGGGATGGCAGAACAGTATCCGCCACAACCTGTCACTCAACGAATGCTTTGTCAAGGTGCCCCGCGATGACCGCAAGCCGGGCAAGGGCAGCTACTGGACGCTGGACCCCGACTGCCATGACATGTTTGAGCATGGCAGCTTTCTGCGCCGCCGCCGGCGCTTCACCCGACGGGCAGGTGCTGAGGGCACCAAGGGCCCCACCAAAGCGCGCCGTGGACCCCTCCGAGCCACCAGCCAGGACCCAGGAGCCCCTGACGTCGCAGCTAGCAGACAGTGCCCATTCCCGCCGGAGCCACTGGAACCCAAGGGCCTAAGCTATGGGGGTCTGGTGGGGGCCTTGCCAGCCAGCATGTGCCCGGCCACCACCAATGCCAGGCCTCAGACACCCTCAGAGGCCAAGGAGATGCCCACTCCCAAGGCTGCAGGCCCAGGGGAGCTCCCTGTGGCCACCTCGTCTTCCTCGTGCCCTGCTTTTGGCTTTCCCACCAGCTTCTCTGAGGCTGAGGGGTTTAGCAAGGCCCCTGCACCCATCTTGACCCCCGAGGCCACCATCGGGAGCAGCTACCAGTGCCGGCTGCAGGCGCTGAATTTCTGCATGGGGACTGACCCAGGACTGGAGCACCTCTTGGCCTCAGCAGCCCCCTCCCCTGCACCATCCACCCCTCCAGCCTCCCTCCGGGCCCCGCTGCCCCTGCCAGCTGACCCCAAGGAACCCTGGGTTGCAGGCAGCTTCCCTGTCCAGGGAAGCTCCAGCTACCCACTGGGGCTGACCCCCTGCCTGTACCGGACGCCAGGAATGTTCTTCTTTGAGTGAAGGCCAGCCGGCCTCAGGCCGTCCCTGCATAGCCCCTGCCAACTACGCCCTCCAGGTTGAGCCTGACTCTGGGATCTGGGGAGCTCTCAAAGGATGCGGGCCTGGCAAGCTCACGACAGCTGGGACAGGAAGCCAAGATTGCAGCGGTGAACACTCAGCCAGCCCTAGGGCCTCTGGACAGACTTGGGGGTGAGGGGAAGCAGGGCCCCCTGGGGATTTACTCTGTGGCTCTCAGGGCCAATAAAGCCAGTGTGATGATGAGGGTCAGTCTGCTGGATGGTTGGCAGCTGAATACCAGGACGCCTGGTTCCATGTAGAGTGGGTGTGGTTGTGGGGTTGAATGGCAGGGGCAGGGGACCCCGGATATAAATGTCCCACATGAGGCTCTCTACTGCTGGGGACTTTCTCAGGGGGTTTCCCAGGAAGGCAGAGGAAAGGGGAGCAGGGAGTGGGAGAGAGAGAGAGGAGGGATGTAATCAGGAGAGATGCCCAGCAGGGTTGGGGCTGGGCGGCACTGGGTGGCTGACCCTCCAGCCCCAAAGTCTGTGACTTGGCTCCCTCATGATGAGCAGCCACAATACCTGTGACCATTTGTGGTCTTTATCCTGTCCTGGGCTCATATATATCTTCTCATATATATATCTATCTATCTGTGTGGTACAGAATGTTATCCCCATTTTACAGTGGTGGAAACTTGAGGCTTAAAGAATTCGGGGCTCACTAGCTCTGGCTCCAAATGGAAAATGAGTGGCAGAGAAGAA

Gray: FOXS1 gene

Blue: STAT1/STAT2-binding element (ISRE)

Yellow: STAT1-binding element (GAS)
