# Supplementary material for: Endometrial factors similarly induced by IFNT2 and IFNTc1 through transcription factor FOXS1
Source: PLoS One. 2017 Feb 15;12(2):e0171858. doi: 10.1371/journal.pone.0171858 (PMC5310909; doi:10.1371/journal.pone.0171858)
Supplement: S3 Fig — (DOCX) [file pone.0171858.s003.docx]

**S3 Fig. The amino acid sequences of IFNT2 and IFNTc1**

> IFNTc1: ENSBTAG00000022303

MAFVLSLLMALVLVSYSPGRSLGCYLSENHMLGARENLRLLAQMNRLSTHSCLQDRKDFGLPWEMVEGDQLQKDQAISVLHEMLQQCFNLFHTEHSSAAWNTTLLEQLCTGLHQQLDDLDACLGQVMEEKDSALGRMGPILTVKKYFQGIHVYLKKKEYSDCAWEIVRVEMIRALSSSTSLQERLRKIGGDLNSS*

　　　　　　 　　 (Casein kinase 2 phosphorylation domain)

> IFNT２: ENSBTAG00000034285

MAFVLSLLMALVLVSYGPGRSLGCYLSEDHMLGARENLRLLARMNRLSPHPCLQDRKDFGLPQEMVEGNQLQKDQAISVLHEMLQQCLNLFYTEHSSAAWNTTLLEQLCTGLQQQLEDLDACLGPVMGEKDSDMGRMGPILTVKKYFQGIHVYLKEKEYSDCAWEIIRVEMMRALSSSTTLQKRLRKMGGDLNSL*
